# Unpacking racism during COVID-19: narratives from racialized Canadian gay, bisexual, and queer men

**DOI:** 10.1186/s12939-023-01961-z

**Published:** 2023-08-08

**Authors:** Cornel Grey, Ian Liujia Tian, Shayna Skakoon-Sparling, Emerich Daroya, Ben Klassen, David Lessard, Mark Gaspar, Jad Sinno, Jordan M. Sang, Amaya Perez-Brumer, Nathan J. Lachowsky, David M. Moore, Jody Jollimore, Trevor A. Hart, Joseph Cox, Daniel Grace

**Affiliations:** 1 Western University, London, Canada; 2 University of Toronto, Toronto, Canada; 3 Toronto Metropolitan University, Toronto, Canada; 4 University of Guelph, Guelph, Canada; 5 Community-Based Research Centre, Vancouver, Canada; 6 McGill University Health Centre, Montréal, Canada; 7 BC Centre on Substance Use, Vancouver, Canada; 8 University of Victoria, Victoria, Canada; 9 BC Centre for Excellence in HIV/AIDS, Vancouver, Canada; 10 Canadian AIDS Treatment Information Exchange (CATIE), Toronto, Canada

**Keywords:** Critical race theory, Racism, Sexuality, Anti-asian racism, Anti-black racism, Gay and bisexual men, COVID-19

## Abstract

**Objective:**

Epidemics impact individuals unevenly across race, gender, and sexuality. In addition to being more vulnerable to COVID-19 infection, evidence suggests racialized gender and sexual minorities experienced disproportionate levels of discrimination and stigma during the COVID-19 epidemic. Drawing on Critical Race Theory (CRT), we examined the experiences of gay, bisexual, queer, and other men who have sex with men (GBQM) of colour facing discrimination during COVID-19.

**Design:**

Engage-COVID-19 is a mixed methods study examining the impact of COVID-19 on GBQM living in Vancouver, Toronto, and Montréal, Canada. We conducted two rounds of qualitative interviews (November 2020 to February 2021, and June to October 2021) with 93 GBQM to explore the evolving impact of COVID-19 on their lives. Transcripts were coded using inductive thematic analysis. Data analysis was conducted using Nvivo software.

**Results:**

Fifty-nine participants identified as Black, Indigenous, and/or a Person of Colour (BIPOC). These GBQM of colour described multiple experiences of discrimination during COVID-19. Although participants did not report experiences of discrimination based on their sexual identity during COVID-19, we found that experiences of racism affected how they were treated within their sexual networks. Experiences of racism were most often reported by East Asian and Black GBQM. These participants faced racism in public and online spaces, primarily in the form of verbal harassment. Several participants were also harassed because they wore face masks. Verbal abuse against GBQM of colour was largely prompted by racist discourses related to COVID-19.

**Conclusion:**

Racism remains a pernicious threat to the well-being of GBQM of colour. CRT highlights the importance of assessing how sexualized and gendered discourses about race shape the experiences of GBQM of colour navigating multiple epidemics like COVID-19 and HIV. These pervasive discourses unevenly affect racial and sexual minorities across multiple epidemics, and negatively impact health outcomes for these populations.

## Introduction

Several scholars have framed the convergence of racism and COVID-19 as a ‘twin pandemic’ [1]. For some, the combination of racism and COVID-19 echoes previous research on race and the HIV epidemic, specifically the disproportionate impact of viral infectious disease epidemics on racialized communities [2, 3]. This article provides a more nuanced understanding of COVID-19 and racism by considering racism as both a structural and interpersonal problem [4]. In this article, we employ Critical Race Theory (CRT) as an analytical lens to unpack the historical, social and political conditions that uniquely affect how racialized gay, bisexual, queer, and other men who have sex with men (GBQM) have experienced the COVID-19 epidemic. We employ CRT to understand how racism, as a structural reality, comes to shape the lives of GBQM of colour during and beyond COVID-19. For instance, in the United States (U.S.), a higher concentration of COVID-19 cases, severe illness, and death have been recorded among Black, Indigenous, racialized, and immigrant communities; often attributed to race-based distribution of social inequality such as crowded housing conditions, economic precarity, and infrastructural neglect [5].

In Canada, where the current study was conducted, race-based data are not systematically collected with COVID-19 infection rates, making it difficult to assess and understand the health risks and racial inequalities during the COVID-19 epidemic [6]. In a recent Canada-wide study, Choi and others examined the association between communities’ demographic composition and the number of COVID-19 infections. As might be expected, they found the highest infection among communities with higher concentrations of Black and/or low-income residents [7]. In this paper, we examined the relationships between racism, health, and the COVID-19 epidemic using CRT as a framework. Our objectives were to examine how white supremacist discourses in settler-colonial countries like Canada lead to culturally specific forms of discrimination that negatively impact the lives of GBQM of colour. Put differently, how did COVID-19 affect how and when GBQM of colour experience discrimination in their everyday social and sexual lives?

## Theoretical Framework

This article treated racism not as an epidemic but as the background for the unfolding of epidemics. We deployed CRT to unpack the underlying and historic social and political conditions that uniquely affect how racialized GBQM have experienced the COVID-19 epidemic. Emerging from legal studies in the 1970s, CRT refers to a broad set of interdisciplinary frameworks and techniques that forefront the ways that racism structures the social worlds in which individuals are nested, including health outcomes, exposure to discrimination and stigma [8–10]. CRT researchers center the experiences of marginalized communities when analysing public policies. They understand race as a socially constructed phenomenon, recognize that racism is a normalized part of everyday life for racialized people and as a result, it can be difficult to measure simply using observable empirical data [11]. CRT advocates for more narrative-based methods, such as sharing experiences through storytelling [8, 12]. In highlighting the experiences of GBQM of colour in Canada during COVID-19, we aim for this work to attend to the inseparable experiences of racism, sexual life, and COVID-19 for GBQM of colour. And we urge public health practitioners and scholars to move towards understanding how racism, gender, and heteronormativity structure unevenly distribute life chances and well-being beyond COVID-19.

We mobilize ‘intersectionality,’ one of CRT’s key theoretical concepts, to understand how participants’ experiences of racism are shaped by interlocking and/or overlapping social categories like gender, sexuality, race, class, disability and citizenship status that can create unique and specific vulnerabilities to harm [13]. Beyond a narrow emphasis on sexual identities and behaviours, our analysis showed how racism shaped the sexual lives of GBQM of colour in Canada during the COVID-19 outbreak. There have been few public health studies that look at how COVID-19 has affected the sexual health and experiences of LGBTQ+ (Lesbian, Gay, Bisexual, Transgender, Queer, and other people who are sexually and gender diverse) people of colour. This research study offers a Canadian perspective, attending to how racism, COVID-19, HIV status, gender and sexuality intersect in our respondents’ lived experiences. We follow CRT’s guide to explore how multiple structures intersect to: (1) produce unequal health outcomes; (2) differential experiences of both extreme and everyday racism.

We further CRT’s critical lens on the biomedicalization and naturalization of socially constructed differentiations such as race [14]. Following CRT’s examination of race as a socially constructed notion rather than pre-determined biological characteristics of individuals, we unravelled how racism affects people’s lives in ways other than viral infections and unequal mortality and morbidity outcomes. Although it is crucial to account for the social-demographic groups that are more severely impacted by COVID-19, it is equally important to understand how racism structures individuals’ decisions, experiences, feelings, attitudes, and perceptions of safety, health, and medical authority [15]. CRT analysis asks, for instance, why one fears going to the hospital or clinic, or where and when one feels unsafe to enter public spaces, such as subways or supermarkets, because of the presence of police and the possibility of racist harassment. For example, one study mobilized CRT to examine poor engagement along the HIV care continuum among Black and Hispanic people living with HIV in the United States [16]. A CRT framework provided a lens through which they could understand how structural racism, legacies of mistrust and exclusion affect racialized communities’ perception of healthcare institutions today.

This paper furthered CRT’s attention to historical contexts as it pertains to racism and the ongoing pandemic. For CRT scholars, racism is not a personal attitude but embedded in social structures, policies and institutions. Although our participants are of multiple ethnic and racial backgrounds, this article focuses on Black and East/Southeast Asian residents of Canada because of the heightened media coverage of anti-Black and anti-Asian racism during COVID-19, particularly during the first year of the epidemic. In many ways, the anti-racist movements we are witnessing seem to be exceptional to our times. Yet CRT teaches us that racial formations have always been historical.

During public health crises, Black, Asian and Indigenous peoples are often portrayed as vectors of disease [17]. In North America specifically, anti-Asian racism during COVID-19 reproduced the ‘yellow peril’ discourses that were prevalent in late 19th century Canada and the U.S [18]. Such discourse has associated East Asian people with disease and portrays them as dangerous virus carriers from a backward, ‘Oriental’ East [19]. These ideas were key reasons behind legislating exclusionary laws such as the Chinese Exclusion Act (1923–1947). Such racist representations continue in contemporary contexts despite the repeal of these acts, and were exacerbated during COVID-19 because the first documented outbreak was in China [20]. Our current study used CRT to contextualize anti-Asian racism during the COVID-19 epidemic not as an individual act of hate, but as part of a longue durée of Canada’s structural exclusion of Asian migrant labour and fears related to imagined ‘yellow peril.’

Media coverage of anti-Black racism and Black Lives Matter protests during COVID-19 have reignited conversations about the various forms of state-sanctioned violence experienced by Black people. Anti-Black racism has a long genealogy rooted in slavery, settler colonialism, and racial capitalism [21]. Without considering the historical contexts of anti-Black racism, public health measures can become harmful, rather than protective, for Black communities. In Canada, gendered anti-Black racism produced multiple structures of oppressions for Black women who work and are family and community caretakers [22]. Among Black residents in Canada, anti-Black racism intersected with immigration status, exposing migrant Black people to multiple axes of health inequalities such as impeded access to healthcare and concerns around vaccination [23].

By pointing out the historical contexts of various forms of racism, this article explored how marginalized sexual and gender identities are imbricated in GBQM’s experiences of racism. Both Asian and Black identities need to be considered in relation to queerness if we are to account for the complex yet interlocking relations and histories between racism and diseases for GBQM of colour.

This research contributed to ongoing research on sexual minorities and the COVID-19 pandemic. In a recent U.S. study, Sears, Conron and Flores examined nationwide data from Axios-Ipsos, and found that fewer LGBTQ + adults trust the federal government and pharmaceutical companies, compared with non-LGBTQ + respondents [24]. Within the heterogeneous category of LGBTQ+, Sears and others concluded that adults of colour are more likely—compared with their white counterparts—to report experiencing economic difficulties during COVID-19 and knowing someone who died of the virus.

We examined the intersecting effects of COVID-19 and racism for diverse GBQM of colour, which has largely been neglected in existing work. We paid attention to participants’ narratives of experiences with public health institutions and mandates, asking how such experiences are produced by intersecting social structures of race, gender, sexuality, and HIV status. Starks and others have already shown how COVID-19 has disrupted access to HIV care and treatment services in the U.S., putting GBQM of colour who are also living with HIV at higher risk than other members of the LGBTQ + community [25].

## Method

### Research Design

The data presented here are derived from the larger mixed-methods Engage COVID-19 study which examines the impacts of the COVD-19 epidemic on the social and sexual lives of GBQM living in Montréal, Toronto, and Vancouver [26–28]. Engage COVID-19 began in September 2020 and is part of an ongoing multi-city cohort study examining HIV, STBBIs and the sexual health of GBQM in Canada [29–31]. We conducted 93 in-depth qualitative interviews over two rounds with GBQM living in Montréal (n = 30), Toronto (n = 33) and Vancouver (n = 30). Participants from the larger prospective cohort study were contacted to ask if they would be willing to participate in an interview about how COVID-19 has affected their lives (including experiences of discrimination). The first round of interviews (n = 42) was conducted between November 2020 and February 2021. The second round of interviews (n = 51) took place between June 2021 and October 2021 with new participants from the same cohort study. Overall, 59 participants identified as Black, Indigenous and/or a Person of Colour (BIPOC).

### Recruitment

Maximum variation sampling was used to capture the diverse experiences of GBQM during COVID-19 [32]. We sought to recruit participants along four key factors: age, ethno-racial background, gender, and HIV status (see Table 1). In recognition of the uneven impact of COVID-19 on racialized communities, we set out to recruit a sample with at least 60% of participants identifying as BIPOC, with at least 10% identifying as Indigenous and 20% identifying as Black. To understand COVID’s impact at different life stages, we set respective targets of 20% and 25% percent for GBQM 55 years of age and over and 25 years of age and under. We set a target of at least 10% for trans men and 20% for participants who identified as bisexual. Finally, we set a sample target of 20% for men living with HIV. Participants were then identified based on quantitative data gathered from the Engage cohort study and contacted for a qualitative interview. Research ethics approval was provided by the research ethics boards of the University of Toronto, Ryerson University, the University of Windsor, Research Institute-McGill University Health Centre, the University of British Columbia, the University of Victoria, and Simon Fraser University.

### Data collection

Owing to restrictions put in place by provincial governments in Canada to limit the spread of COVID-19, all interviews were conducted virtually using MS Teams [33]. A semi-structured interview guide was developed by the research team, in collaboration with community engagement committees (CECs) in Montréal, Toronto and Vancouver. Members of the CECs included service providers and representatives from the GBQM community living in each city. The CECs provided feedback and direction on study design, study implementation, and analysis of data. Interviews in Montréal were conducted in English or French, according to participants’ preferences. Interviews in Toronto and Vancouver were conducted in English only. Participants were given time to review the consent form in advance of the interviews. Participants provided written and verbal consent. The average length of interviews was 81 min, which were.

documented using a recording device and/or through the MS Teams platform. Participants were given the option to have their camera on/off during the interview. Interviews conducted in Montréal and Vancouver were conducted by two white researchers. Interviews conducted in Toronto were completed by a Black researcher. All interviewers identified as GBQM. All three interviewers have resided in the city where they conducted interviews for several years and their understanding of local geographies and political tensions allowed them to ask targeted follow-up questions and probes during interviews. Their local knowledge of each city and its communities enhanced our analysis of participants’ descriptions of racist harassment. Participants were given the choice to turn on/off their cameras during the interview. Participants received a $50 CAD honorarium for their time.

The interview guide had seven key domains: (1) introductions, socio-demographics, and rapport building; (2) experiences and risk factors for COVID-19, including understanding and uptake of public health and community COVID-19 messaging; (3) effects of the COVID-19 epidemic on finances, work, and everyday life; (4) access to health services during COVID-19; (5) sexual health and sexual decision-making; (6) psychological impacts, mental health, and substance use patterns; (7) additional issues of concern and closing reflections. Participants were asked whether they had experienced and/or witnessed discrimination related to COVID-19. In section two, they were also asked if they experienced any discrimination when accessing health services (e.g., testing for COVID-19). For many participants, discrimination cut across several interview domains as they conveyed their experiences of racism during provincial lockdowns and its impact on their mental health, encounters with Anti-Asian rhetoric in news media, and concerns about the prevalence of racism within healthcare institutions.

This analysis included 59 participants with a mean age of 36 years (range: 20–64 years). Approximately 14% (13.6%) of participants identified as Black, most of whom resided in Toronto. Almost a quarter (23.7%) of participants identified as East Asian, half of whom resided in Vancouver. Most participants identified as cisgender (86.4%) and gay (78.0%). About one fifth of participants (18.6%) reported living with HIV (See Table 1).

**Table 1:** Sociodemographic Characteristics of Study Participants (N = 59)

	Montréal (n = 17) n (%)	Toronto (n = 21) n (%)	Vancouver (n = 21) n (%)	Overall (N = 59) n (%)
*Age in years*				
< 25	3 (17.6)	2 (9.5)	0 (0.0)	5 (8.5)
25–30	2 (11.8)	7 (33.3)	7 (33.3)	16 (27.1)
31–40	8 (47.0)	8 (38.1)	7 (33.3)	23 (39.0)
41–50	1 (5.9)	3 (14.3)	2 (9.5)	6 (10.2)
51–60	2 (11.8)	0 (0.0)	4 (19.0)	6 (10.2)
60+	1 (5.9)	1 (4.8)	1 (4.8)	3 (5.0)
*Ethno-Racial Identity*				
Black	3 (17.6)	4 (19.0)	1 (4.8)	8 (13.6)
East Asian	1 (5.9)	6 (28.6)	7 (33.3)	14 (23.7)
Indigenous	1 (5.9)	0 (0.0)	1 (4.8)	2 (3.4)
Latin American	2 (11.8)	3 (14.2)	1 (4.8)	6 (10.2)
Middle Eastern	1 (5.9)	1 (4.8)	1 (4.8)	3 (5.0)
Mixed Race/Ethnicity	8 (47.0)	6 (28.6)	8 (38.0)	22 (37.3)
South Asian	0 (0.0)	1 (4.8)	2 (9.5)	3 (5.1)
Southeast Asian	1 (5.9)	0 (0.0)	0 (0.0)	1 (1.7)
*Gender Identity*				
Cisgender man	14 (82.4)	19 (90.5)	18 (85.7)	51 (86.4)
Trans/ Non-binary/Genderqueer	3 (17.6)	2 (9.5)	3 (14.3)	8 (13.6)
*Sexual Identity*				
Bisexual	3 (17.6)	1 (4.8)	1 (4.8)	5 (8.5)
Gay	12 (70.6)	17 (80.9)	17 (80.9)	46 (78.0)
Pansexual	0 (0.0)	0 (0.0)	1 (4.8)	1 (1.7)
Queer	2 (11.8)	3 (14.3)	2 (9.5)	7 (11.8)
*HIV Status, Self-reported*				
HIV-Negative	14 (82.4)	18 (85.7)	16 (76.2)	48 (81.4)
Living with HIV	3 (17.6)	3 (14.3)	5 (23.8)	11 (18.6)

### Data analysis

BIPOC participants’ (n = 59) descriptions of their ethno-racial backgrounds were collected during the interviews. When asked how they would describe their ethno-racial background, most participants identified as “mixed” or described themselves as belonging to multiple ethno-racial communities, including participants who identified as “Black and Indian,” “white and Asian,” “white and American Indian,” “English and Chinese.” Here, we use “Mixed Race/Ethnicity” not to reaffirm race as a biological category, but to capture participants’ descriptions of their histories and the multiple communities to which they belong. Our analysis was also attentive to how mixed-race participants understood their experiences of discrimination in relation to their perceived relationship to a particular community (e.g. whether they ‘looked’ East Asian). Data from the interviews were transcribed verbatim and transcripts were subsequently reviewed for accuracy. Ten out of 17 Montréal interviews were conducted in French. Any discrepancies were resolved by interviewers who compared transcripts to the original audio recording. Transcripts were imported into QSR Nvivo 12 software and coded using inductive thematic analysis [34].

Data analysis was led by the two lead authors of this manuscript, one Black and the other East Asian. Both drew on lived experience of racism in Canada as well as their expertise in critical race theory during the reflexive interpretation of these narrative accounts. Our approach to coding and analysis was three-fold. First, we reviewed the transcripts and discussed interviewers’ reflections following each interview. Second, overarching codes were developed by GBQM of colour on the research team to organize key data from the interviews into coherent sections (e.g. anti-Black racism, anti-Asian racism). CRT informed the data analysis process by alerting us to the various forms of everyday racism that GBQM of colour in our sample face. Critical race theorists note that racism is often subtle, not in its impact on peoples’ lives, but in terms of how it has been normalized and made invisible. This resulted, for example, in GBQM of colour being unsure if they experienced racism. A CRT approach demanded that we not only examine participants’ conscious descriptions of discrimination during COVID, but also interrogate their everyday experiences in relation to discourses about race relations in Canada and the nation’s settler-colonial history. Finally, we reassessed and refined significant themes, and highlighted trends in the data to help us understand the specific experiences of racism during COVID-19 as detailed by GBQM of colour in our study.

Across all interviews, participants expressed concern about how different groups of people were treated (and might be treated) during the COVID-19 pandemic. ‘Discrimination’ emerged early on as a high-level code. Specific forms of discrimination (i.e., homophobia, anti-Black racism, anti-Asian racism) were developed as sub-codes. At the same time, GBQM made multiple comparisons to previous public health crises where Two-Spirit, lesbian, gay, bisexual, transgender, and queer (2SLGBTQ+) and/or racialized communities were targeted (i.e., HIV, SARS, Ebola, Swine Flu). We arrived at our two primary themes through an examination of GBQM’s descriptions and fears about the way COVID-19 might affect their communities based on past experiences of discrimination and an observation of how COVID-19 activated and repackaged familiar racist discourses during the pandemic.

## Results

Two main themes were identified through our analysis of discrimination, marginalization, and resource access: (1) anticipated impacts of COVID-19 on racialized communities and (2) manifestations of racism during COVID-19. These two interrelated themes are further divided into subthemes. These domains captured participants’ concerns about the impact of COVID-19 on minority communities, and participants’ experiences with various forms of discrimination such as anti-Asian and anti-Black racism.

### Anticipated impacts of COVID-19 on racialized communities

#### Racialized neighbourhoods and public health interventions

Participants’ identification of vulnerable populations during COVID-19 accounted for multiple social categories and drew on their understanding of how racism and class segregation operate in their cities. GBQM were also attentive to the political and geographic contexts that impact how and when social identities and locations matter as shown below:

I think the big one, for me, in Montréal, was housing. And a lot of my friends like have been in very, very precarious housing situations, not just due in part to the pandemic. Yeah. Like rent was really hard for a lot of people. (20s, Mixed Race, Queer, Montréal)

[E]veryone was like, “Oh, look at NDG.^1^ Oh, look at Montréal-Nord.” It’s places predominantly that are poor, Black and Brown communities, poor communities, immigrant communities, and all it was, was a big focus on, “Don’t go to NDG, it’s like the worse zone in Montréal”, instead of being like, “Wow, people in NDG are really being forced to work”, when you can walk 10 more minutes into Westmount^2^ and everyone in Westmount was hanging out just fine and treating like the pandemic was not even happening. (20s, East Asian, Queer, Montréal)

In Metro Vancouver, a participant noted that high COVID-19 cases in one municipality produced racist and moralistic responses about health literacy and COVID-19 prevention.

[O]ne of the interesting news points that became quite publicized was how like in Surrey, which is [a] predominantly South Asian community, we were seeing spikes in COVID because there was intergenerational households, the questions of like knowledge translation and like literacy and like understanding a western public health approach through very a very medicalized lens…seeing the rise of racist rhetoric that came alongside that too of like all those people out in that community just like should know better, don’t know what they’re doing or what have you and very much like blaming the communities for like the lack of like a better public heath approach. (30s, South Asian, Vancouver)

Participants in our study noted the disproportionate impact of the COVID-19 crisis on racialized communities, those who face housing instability and live in under-resourced neighbourhoods. As we can see from the Vancouver participant quoted above, racialized communities were also being blamed for the impact of COVID-19 within their communities, positioning them as both agents and victims of COVID-19 transmission. Communities of colour, particularly those with largely working-class families were treated both as vulnerable to, and blameworthy for, COVID-19. Our participants’ descriptions suggest that there were common-sense understandings of these communities as being different from other spaces in the city. Much like how ‘China’ was constructed as a country, an ethnicity, and as COVID-19 personified, some members of the public associated particular neighborhoods with specific racialized communities. The communities were also imagined as naturally engaging in behaviours that undermined public health and safety. GBQM of colour in our study understood the implications of these discursive practices and as a result, some participants anticipated discrimination when interacting with other people like healthcare professionals.

#### Fear of COVID-19 care given experienced racism in healthcare settings

GBQM of colour in our study narrated past experiences of racism within healthcare settings and reflected on COVID-19 related care trajectories. These participants demonstrated an awareness of how racism is embedded within institutions and how that might affect their healthcare experiences during COVID-19. When asked if he had any concerns about COVID-19 on his health, this Montréal participant focused not on his current health status, but rather shared concerns about racism from healthcare providers.

I wonder if I would get less sympathy from doctors and healthcare professionals, or even if, you know, I have to go to fill up a prescription at the pharmacy or I have to go see the doctor, like I wonder if people would treat me differently because they’re like, “Oh, he’s Chinese, he has COVID.” (20s, East Asian, Queer, Montréal).

When describing the impact of COVID-19 in his life, including the death of family members, this participant conveyed the difficulty of not being able to visit ill relatives at the hospital.

I know from my own story, I was lucky because half [of] my family is white, the other half is Black, and I had white family members advocating for me, and it fully transformed my outcome in the health system. And until they actually did that it was quite – it was really, really bad. (30s, Black, Pansexual, Vancouver)

During COVID-19, GBQM of colour struggled to reconcile risks to their health as they reflected on how they would be treated when seeking care during COVID-19. They were attuned to discourses about race that they felt would result in differential treatment based on how their bodies were perceived.

### Manifestations of racism during COVID-19

#### Anti-asian racism during the first wave

East Asian GBQM interviewed in the first round of interviews described multiple experiences where they were harassed or discriminated against based on their perceived race. In some cases, these encounters manifested as heated confrontations. In other instances, participants observed individuals intentionally keeping their distance, for perceived fear of acquiring COVID-19. When asked whether he had experienced and/or witnessed any racism or discrimination related to COVID-19, the participant below notes that anti-Asian racism was commonplace before the COVID-19 epidemic began; it merely increased within the context of COVID-19:

[T]he racism I’ve received, yes, increasing. Like, some people, random people, will call you some really bad names when you’re walking on the street, for no reason. That appeared a few times... Yes. Mostly, like, some racial slurs from subway or some random people…. I have experienced like this a few times before but after COVID, especially in the beginning of the year [2020], it’s like I would say it’s like three times maybe a month.

(20s, East Asian, Gay, Toronto)

Participants across all three cities described incidents in which they were singled out in public and verbally assaulted during COVID-19.

[T]here are two white men yelling at me at 6:00 am, and then just following me and yelling at me. And then – because it’s early in the morning, and there is nobody around. And then, I’m a little bit scared. Then, when I take the bus, get into the bus, I tried to scare him back, and then using my cell phone to pretend that I’m taking a photo of them, and then they still keep yelling outside the bus. (60s, East Asian, Gay, Vancouver)

Some guy came to me and say that “your people have done a bad thing to the world.” But he didn’t know that I wasn’t born in China and I’ve never left Canada for 14 years. (60s, East Asian, Gay, Toronto)

East Asian GBQM described multiple incidents where they were harassed in public by strangers without provocation. Across all city contexts, their bodies were perceived to be a threat to public safety and so were subject to racist violence. Although not all these incidents took place in crowded settings, our participants did not describe any case involving bystander intervention.

#### COVID-19 prevention and racism

Provincial governments in Canada implemented mask mandates at various points during COVID-19. While the length and scope of these guidelines varied by province, masks were generally required when accessing a service indoors. Although experiences of racism were primarily reported by East Asian and Black GBQM, one Latino participant attributed a racist insult he received (i.e., someone shouted ‘slave’ at him as they passed by), to him wearing a mask while running errands. Some East Asian GBQM in our sample also shared that they (along with family members) wore face masks before COVID-19 to prevent other people getting sick when they were ill. Within the context of COVID-19, however, face masks became a compounding factor for the racism experienced by these Vancouver participants interviewed in the first round.

I don’t know. I don’t know. He just keep on yelling at me and I don’t know – at that time, I did wear mask, yes. I don’t know whether on wearing mask or because I’m Asian. Because they thought that the virus come from China or from Asia, and that’s why they thought “you guys bring the virus to us.” (60s, East Asian, Gay, Vancouver)

[W]hen we go out, even we have masks at home, we are scared to wear. Because people may discriminate us or think that oh, how come you are sick and you still running around? (30s, East Asian, Gay, Vancouver)

For some Black GBQM, wearing a face mask in public meant experiencing increased surveillance in their day-to-day lives. Black GBQM demonstrated an awareness of how their bodies would be perceived by others as threatening. They reflected on the meaning of these interactions within the context of COVID-19.

[W]ith wearing masks and being a Black man in a mask, you don’t have other ways of communicating to people and so they, you are judged by the colour of your skin and nothing else because you’re pretty much covered everywhere else. So that’s been, I mean but that’s kind of always been there, the Black man living in these spaces but I’m more aware of it I guess now because I’m limited in my way of communicating.

(30s, Black, Gay, Toronto)

I’ve had instances with neighbours where they don’t recognize me because of my mask so they see my hair because it’s curly and it’s bigger, and they might see some facial hair on the sides and, like, two weeks ago I was just sitting in front of my place and one of my neighbours who I’ve met many times didn’t recognize me but came up to me and asked if I was waiting for someone. I said no, I wasn’t, I was just sitting in a common area outside following COVID protocol. And they told me to leave. Basically, their words were, “get off the property.” (30s, Black, Pansexual, Vancouver)

For both East Asian and Black GBQM, the adoption of face masks as a public health mandated COVID-19 prevention strategy resulted in increased exposure to racism.

#### Sexual racism and a politics of desirability

The impact of COVID-19 on the forms of racism experienced by GBQM of colour extended beyond anxieties about acquiring the coronavirus and its impact on their health. GBQM of colour expressed concern that they were seen as (potential) vectors of COVID-19 transmission, and that perception had a negative impact on their sexual lives.

I found [camming]^3^ a really hard space to break into. I think maybe now would be different, but again, like at the beginning, because I was Chinese, a lot of people were not really loving seeing me online, because I think people were like, “Oh, another Chinese person.” Like, they were feeling like they were being reminded of COVID if they saw me, whatever... A lot of people would join my show just to write a bunch of hate...sometimes they’re there to, like, antagonize you, because maybe they’re more of like a sadist, sort of, sexually, so for them, it’s sort of hot for them to be racist.

(20s, East Asian, Queer, Montréal)

When asked if he had any concerns about COVID-19, the participant below stated that he was worried that COVID-19 would lead to more discrimination in the gay community. He explained that before COVID, white men did not want to pursue a relationship with him. He shared his anxieties regarding the impact of racism on his dating life during COVID-19.

[I’m concerned that guys are] going to say oh, you’re from Latin America, probably Latin America doesn’t have – people in Latin America has no, does not have the vaccine so I don’t want to meet with you, I don’t want to be around you… because I remember when, before, when I travelled to Colombia, my country. So, and I meet my friends here, they say that, “oh, you just came from Colombia but didn’t you bring anything or something like that? Like coronavirus or just something.”

(40s, Latino, Gay, Toronto)

These examples provide a brief snapshot into the sexual lives of GBQM of colour during COVID-19, alerting us to a politics of desirability where some individuals are defined as ‘clean’ (i.e., white) and ‘unclean’ (i.e., racialized, immigrant). Although our analysis here has primarily featured the experiences of East Asian and Black men, the experiences of the Latino participant quoted above alerts us to how GBQM are making decisions about partner selection and sex during COVID-19.

## Discussion

Findings underscore the pervasive and multi-scalar impacts of racism during COVID-19. This included an understanding of how structural racism cultivates conditions where racialized neighbourhoods are chronically under-supported and people of colour are more likely to experience barriers to healthcare. We also identified and examined the subtle ways racism affects GBQM of colour’s social and sexual lives during COVID-19. Participants in our study often described racism as ubiquitous, as many GBQM of colour were used to hearing racist remarks or being told that they do not belong in a given space. Within the context of COVID-19, however, the proliferation of bold-faced racist harassment created immediate and acute safety risks for people of colour, as evidenced by the experiences reported by the GBQM in our study. This included, for example, anxieties regarding the quality of healthcare they might receive (which may lead to hesitancy to access health services), as well as the psychological distress caused by experiencing prejudice and harassment [35].

Despite promoting multiculturalism as a uniquely Canadian value in recent decades, Canada has legitimized anti-Asian and anti-Black racism [36–41]. Institutional forms of racism shape Canadian culture, specifically the extent to which Black and Asian communities are seen and treated as full citizens. Our intersectional analysis indicates that gender and sexuality played a role in how GBQM of colour have experienced racism during COVID-19. The media regularly portrays Black men as aggressive and threatening, whereas East Asian men are often portrayed as more feminine and weak, and East Asian GBQM even more so [42, 43]. The construction of East Asian GBQM as particularly feminine in the western imagination may make them more vulnerable to racist harassment, as members of the public may believe they are less likely to respond to provocation. Indeed, East Asian GBQM in our study may have been seen as easy targets, as they reported many regular experiences of harassment. It is noteworthy that the participants who described targeted anti-Asian attacks also belonged to age groups that made them more susceptible to harassment. These participants were either in their early twenties or their sixties, both stages of life where other men might assume they can take advantage of perceived social and political impotence [44].

On the other hand, findings echo literature reporting that Black men must contend with stereotypes depicting them as threatening, a problem that has been exacerbated by public health guidelines regarding the wearing of masks [45, 46]. Such portrayals are the legacy of the ‘Black brute’ stereotype that constructed Black men as animalistic, overly aggressive and dangerous [47, 48]. Black GBQM reported increased surveillance while wearing a mask, even at home. In the case of a Vancouver participant quoted above, his attempt to follow provincial public health guidelines and take care of his health exposed him to a familiar, but nonetheless frightening, threat to his well-being when his neighbour questioned him about being on their shared property. This example of anti-Black racism caused significant stress for the participant, and could have easily escalated to include law enforcement personnel and potentially result in his death [49]. For one of our Black Toronto participants, face masks have changed how he navigates interaction with people daily. The fear that someone might think he is a threat was an ongoing concern for him and created a degree of anxiety in his life as he felt self-conscious about appearing sufficiently personable and non-threatening to those around him.

We also found indications that COVID-19 is uniquely restructuring the sexual lives of many GBQM of colour. Critical race theorists understand that epidemics like COVID-19 magnify, rather than disrupt, systems of power that facilitate discrimination and other forms of exclusion that affect racialized communities. Although everyone experienced limitations to in-person sex following provincial lockdowns, including social distancing requirements, people of colour experience COVID-19-specific sexual racism in both physical spaces and on virtual platforms [50–52]. Coined by Charles Herbert Stember, sexual racism refers to a specific kind of racial discrimination based on sexual and/or romantic desire [53, 54]. Within the context of a potential sexual and/or romantic encounter, an individual is evaluated based on their (perceived) race or ethnicity. Historical narratives, local stereotypes and other public discourses about race all contribute to understandings of sexual desirability. In Anglocentric, settler-colonial countries like Canada, whiteness functions as a form of erotic capital [50]. As a result, Black and Asian GBQM often face significant challenges navigating racism in their sexual and romantic relationships, as well as social relationships within the 2SLGBTQ + community [50, 55–58].

During COVID-19, Several participants shared their concerns about being discriminated against based on their visible or perceived affiliation with a particular racial group. In the examples from the participants in our study, the expectation that a GBQM of colour must prove that they are not COVID-positive was not merely an example of COVID-safe practices. It is grounded in a worldview that understands racialized people as a danger to one’s health. Race, non-normative sexual practices, and diseases are epistemically structured. Whiteness or European-ness is connected to purity, modernity, and hygiene whereas certain racial and sexual differences are coded as perverse, dirty, and disease-laden [59].

In their recent work, Arscott et al. detailed how Black HIV-negative men experienced HIV-related stigma resulting from the racialization of HIV [60]. The higher prevalence of HIV within Black communities and media stereotypes about Black men on the “down low” who infect their partners were cited as some of the reasons why Black GBQM experienced discrimination when pursuing sexual relationships. The hypersexualization of Black GBQM was also found to contribute to stigma, as other men might assume that Black men are promiscuous and more likely to engage in risky sexual practices. Indeed, the racialization of COVID-19 has already taken place [61]. This process affects GBQM of colour differently and unevenly, but it references existing sexual discourses regarding the ‘cleanliness’ and trustworthiness of GBQM of colour. As described by an East Asian participant, COVID-19 is imagined by many as a “Chinese virus” and so potential partners may assume that East Asian people are carriers of the virus based on their (perceived) ethnicity. And although the comments that were directed towards a Latino participant (i.e. ‘bringing back COVID’ from Colombia) had a racist undertone, it is also influenced by negative stereotypes about the sexualities of Latino men [62]. Pervasive stereotypes about Latino men include depictions of them as promiscuous and untrustworthy partners. These racist representations may then inform how the behaviours of Latino GBQM are perceived by potential sexual partners during COVID (i.e., they are more likely to spread COVID because they have multiple sex partners).

Although this study makes an important contribution to understanding the experiences of GBQM of colour during COVID-19, there are a few limitations to be considered. This study would have benefited from being able to recruit more Black and Indigenous GBQM, considering the disproportionate impact of the COVID-19 within those communities in Canada [63, 64]. COVID-related impacts may have also affected Black and Indigenous participants’ capacity to participate in an interview (e.g., work schedules, lack of resources and time). Recruiting Black and Indigenous participants was an explicit priority when we began our interviews, however, both prior failure in the larger cohort study to reach these populations and structural barriers meant these participants could not participate equitably. Our sample is also unable to fully address the extent to which other GBQM of colour (e.g., Latin American, South Asian, Middle Eastern) experienced discrimination over time. In terms of participant recall, the timeline of the interviews may have posed a challenge for some GBQM as reports of racism were particularly pronounced during the first few months of the COVID-19 epidemic, and our interviews took place several months after this time; GBQM in the second round of interviews expressed some difficulty in recounting experiences of discrimination during the earlier months of COVID-19. Nonetheless, we believe that we have gathered sufficient data to develop a nuanced understanding of how GBQM were affected by racism over the course of COVID-19. Research examining COVID-19’s impact on minority populations has primarily focused on racialized communities with little attention to sexuality [3, 23, 64–66]. Importantly, this study fills a gap in the existing literature examining the COVID-19-specific experiences of GBQM of colour during concurrent epidemics.

On July 23, 2022, the World Health Organization (WHO) declared mpox a public health emergency of international concern (PHEIC) [67]. Cases peaked in Canada during June of 2022 with GBQM being among the most affected [68]. Homophobic and racist discourses related to mpox raised questions about lessons learned during the COVID-19 and HIV epidemics as GBQM and people of African descent were subject to various forms of racist violence [69]. Racist stigma was so pronounced that it necessitated the renaming of the virus. The homophobic and racist nature of public discourse during the mpox outbreak disproportionately affected Black queer people. Although the outbreak is considered over, public health officials in Canada have expressed some concern about a possible spike during the summer of 2023. GBQM across the country have been encouraged to get vaccinated for mpox [68]. In light of GBQM of colour’s experiences of racism during COVID-19 and reports of anti-Black racism and homophobic during Canada’s recent mpox outbreak, our call for public health interventions to address the impact of racism on the lives GBQM in Canada remains urgent. Failure to address racism not only has negative implications for the physical and mental health of GBQM of colour, it may also put undue strain on public health resources should GBQM of colour feel pressured to seek out biomedical interventions like vaccines to convey that they are ‘safe’ sexual partners [70]. Sexual health clinics, for example, may face crowds of GBQM seeking vaccination cards not as a preventative measure for infection, but as a mitigation strategy against sexual racism.

## Conclusion

Racism continues to pose a threat to GBQM of colour’s health during COVID-19, lessening appropriate healthcare access and imposing discrimination and mental health burdens. It also poses a significant challenge to GBQM’s social connections as racist and gendered assumptions about individuals’ bodies and behaviours inform how GBQM are treated in their public and private lives. Our findings demonstrate the relevance and urgency of CRT to the study of COVID-19 (and other public health crises like HIV and monkeypox) and the lives of racial and sexual minority populations. It also communicates the need for more comprehensive analyses that examine how COVID-19 affects the sexual and social relationships of marginalized communities. Public health interventions in Canada must anticipate the ways in which racialized and minoritized populations are made targets of stigma during epidemics and prioritize racism as a defining factor in how members of these populations experience and recover from these crises.

## Data Availability

The data used to support the findings of this study are included in the article.

## Footnotes

1 Notre-Dame-de-Grâce (NDG) is a middle-class neighborhood in the Western part of Montréal. It is a highly diverse community, with several Afro-Canadian and immigrant communities rooted there.

2 Westmount is an enclave of the city of Montréal. It is an affluent suburb with predominantly white residents.

3 Camming refers to an online practice where an individual receives payment for performing sexual acts (e.g., stripping, masturbation) via a webcam.
